# Scientific Items

**Published:** 1884-03-20

**Authors:** 


### SCIENTIFIC ITEMS.

**Metallic Cloth.**—A novel invention is about to be commercially utilized, it would appear, on a considerable scale—namely, the production of metallic cloth, which, if desired, can be impressed with a pattern or printed upon. In the manufacture of this curious fabric the metal intended to be used is rendered into small particles, short or long, according to requirements, and in this form is mixed with a sticky material, such as india-rubber. The mixture, as thus prepared, is transferred to goods made from cotton, wool, linen, silk or other textile, after which the fabric is dried and calendered, or dressed, in a suitable manner, so as to produce a smooth, metallic face. The process having thus been carried on to this extent, the cloth can be impressed with a desired pattern by the simple operation of embossing, or the pattern may be printed on its surface in the usual manner. The liquid charged with the metal does not penetrate to the under side, but leaves it quite clean. The finished material is quite soft to the touch and very pliable.

**Enormous Growth of Railway Interests.**—Few realize the enormous growth and present extent and importance of our railway interests. In 1833, fifty years ago, there were 383 miles in operation in the United States; at the close of 1882 there were 115,000 miles. In 1830 there were only thirty miles, and even in 1860 only 30,635 miles of railways. In 1882 the earnings of the railways of the United States could not have been less than $850,000,000, and they probably carried during that year nearly 450,000,000 tons of freight. They employed in some way about 1,750,000 men, and furnished support to at least 8,000,000 persons, not counting the families supported, in whole or in part, by dividends from the earnings. Including in the number of people supported by railways those engaged in the manufacture of railway supplies, we may safely say that the railways furnish support to 9,000,000 persons, or about one-sixth of our entire population.—*Railway Gazette.*

**On the Possibility of making a Cadaver Disappear Entirely by the use of ordinary Sulphuric Acid.**—A. P. Reynard, (Comptes Rendus de la Societe de Biologie) tells us that, in consequence of the attempts made by Prof. Girard to totally destroy the bodies of sheep affected by charbon by placing them in their weight of commercial sulphuric acid, and thus destroy the virus that Pasteur declares cannot be destroyed by burial, he concluded that here was a means which might be employed to destroy the evidence of crimes committed, and made certain experiments to see how far a human body could be so disposed of.

This method could not very well be used in the case of adults, as it would require too much acid and vessels of too large a size; but in the case of abortion or infanticide, unfortunately, nothing is easier than to destroy all traces of the crime by means of sulphuric acid. He placed the body of a new born child, weighing three kilos, in a suitable vessel containing four liters of sulphuric acid. Thirty hours later, without the giving off of heat or odor, the cadaver had disappeared. The best means of disposing of the remaining acid is that which would most readily occur to the mind of the criminal—that is, to throw it into the drainage of the house; the pipes, being made of pottery, are not affected; the acid encounters the carbonate of ammonia from the urines, etc., sulphate of ammonia and carbonic acid is the result, and all trace, not only of the crime itself, but also of the means used to conceal it, is obliterated.—*Jr. Amer. Med. Prog.*

**The Earth More Rigid Than Steel.**——Professor Sir W. Thomson, in his new treatise on natural philosophy, is led, by a consideration of the necessary order of cooling and consolidation of the earth, to infer that the interior of our world is not, as commonly supposed, all liquid, with a thin solid crust of from thirty to one hundred miles thick, but that it is on the whole more rigid than a continuous solid globe of glass of the same diameter, and probably more rigid than such a globe of steel.—*Druggists' Circular.*

**Confirmation of Professor Young's Explanation of the Atmospheric Glow.**—Just as we are on the point of going to press, Nature, for December 20, 1883, comes to hand with several interesting communications on the recent remarkable atmospheric phenomena. Among these is one from Holland, stating that a comparison of the grayish sediment from rain there on the 13th of December with volcanic ashes brought from Java since the great eruption shows that the two are identical in composition. "Both the sediment and the volcanic ash contained (1) small, transparent, glassy particles, (2) brownish, half-transparent, somewhat filamentous little staves, and (3) jet-black, sharp-edged, small grains resembling augite." A geologist in Madrid, also, who analyzed fresh-fallen snow in that locality, states that he has found "crystals of hypersthene, poroxine, magnetic iron and volcanic glass, all of which have been found in the analysis lately made at Paris of the volcanic ashes from the eruption of Java." There could hardly be a more distinct confirmation of the view that the atmospheric phenomena were really due to the Krakatoa eruption.—*Bost. Jour. Chem.*

**The Beauti ul Snow.**—A Swiss scientist, Floegel, is said to have found, in examining the residue from the evaporation of freshly-fallen snow, living infusoria and algæ, bacilli and micrococci, mites, diatoms, spores of fungi (in immense numbers), also fibers of wood, mouse hairs, pieces of butterfly-wings, skin of the larvæ of insects, cotton-fibers, pieces of grass, epidermis, pollen-grains, rye and potatoe flour, grains of quartz, minute pieces of roofing-tiles, with bits of iron and coal.

